# Intra-fractional patient setup error during fractionated intracranial stereotactic irradiation treatment of patients wearing medical masks: comparison with and without bite block during COVID-19 pandemic

**DOI:** 10.1093/jrr/rraa101

**Published:** 2020-11-12

**Authors:** Shingo Ohira, Naoyuki Kanayama, Riho Komiyama, Toshiki Ikawa, Masayasu Toratani, Yoshihiro Ueda, Hayate Washio, Masayoshi Miyazaki, Masahiko Koizumi, Teruki Teshima

**Affiliations:** Department of Radiation Oncology, Osaka International Cancer Institute, Osaka, Japan; Department of Medical Physics and Engineering, Osaka University Graduate School of Medicine, Suita, Japan; Department of Radiation Oncology, Osaka International Cancer Institute, Osaka, Japan; Department of Radiation Oncology, Osaka International Cancer Institute, Osaka, Japan; Department of Radiation Oncology, Osaka International Cancer Institute, Osaka, Japan; Department of Radiation Oncology, Osaka International Cancer Institute, Osaka, Japan; Department of Radiation Oncology, Osaka International Cancer Institute, Osaka, Japan; Department of Radiation Oncology, Osaka International Cancer Institute, Osaka, Japan; Department of Radiation Oncology, Osaka International Cancer Institute, Osaka, Japan; Department of Medical Physics and Engineering, Osaka University Graduate School of Medicine, Suita, Japan; Department of Radiation Oncology, Osaka International Cancer Institute, Osaka, Japan

**Keywords:** Intra-fractional patient setup error, intracranial stereotactic irradiation, bite-block, COVID-19

## Abstract

The immobilization of patients with a bite block (BB) carries the risk of interpersonal infection, particularly in the context of pandemics such as COVID-19. Here, we compared the intra-fractional patient setup error (intra-SE) with and without a BB during fractionated intracranial stereotactic irradiation (STI). Fifteen patients with brain metastases were immobilized using a BB without a medical mask, while 15 patients were immobilized without using a BB and with a medical mask. The intra-SEs in six directions (anterior–posterior (AP), superior–inferior (SI), left–right (LR), pitch, roll, and yaw) were calculated by using cone-beam computed tomography images acquired before and after the treatments. We analyzed a total of 53 and 67 treatment sessions for the with- and without-BB groups, respectively. A comparable absolute mean translational and rotational intra-SE was observed (*P* > 0.05) in the AP (0.19 vs 0.23 mm with- and without-BB, respectively), SI (0.30 vs 0.29 mm), LR (0.20 vs 0.29 mm), pitch (0.18 vs 0.27°), roll (0.23 vs 0.23°) and yaw (0.27 vs 22°) directions. The resultant planning target volume (PTV) margin to compensate for intra-SE was <1 mm. No statistically significant correlation was observed between the intra-SE and treatment times. A PTV margin of <1 mm was achieved even when patients were immobilized without a BB during STI dose delivery.

## INTRODUCTION

The aftermath of the outbreak of the coronavirus disease 2019 (COVID-19) in Wuhan, China, has witnessed the spread of infections from Asia to Europe and the USA. Consequently, the World Health Organization (WHO) has declared COVID-19 a global pandemic. According to the WHO scientific brief, interpersonal transmission of COVID-19 occurs through droplet transmission from coughing, sneezing or talking between people during close contact [1]. Because infective respiratory droplets can transmit the virus through exposed mucosae or conjunctiva, the wearing of a medical mask is one of the primary prevention measures for minimizing the spread of COVID-19 [2].

In this context, Liang *et al*. have reported that cancer patients are more susceptible to infection by COVID-19 than individuals without cancer owing to their immunosuppressive condition caused by malignancy and cancer treatments [3]. As regards radiotherapy treatments, which generally cover several weeks, medical staff need to protect susceptible cancer patients from infection (from staff to patients) during the treatment period. Similarly, medical staff need to be protected from interpersonal transmission (from patients to staff). Additionally, the normally used closed environment for radiotherapy treatments (including shielded treatment rooms with inadequate ventilation in underground locations) may increase the risk of infection. In this regard, after the outbreak, Wei *et al*. studied the patient setup accuracy of cranial and head-and-neck patients immobilized with thermoplastic masks as well as medical masks, and they found that the setup accuracy with the medical mask–thermoplastic mask combination was comparable with that of thermoplastic masks alone [4].

In the context of radiation treatment, we note that patients with brain metastases have historically been treated with conventional fractionated whole-brain radiotherapy (WBRT). Meanwhile, the recently developed stereotactic irradiation (STI) technique allows the accurate delivery of high radiation doses in a few fractions with the application of sophisticated radiation planning, accurate patient immobilization and volumetric image guidance irradiation techniques [5–7]. Thus, STI treatment results in reduced radiation-induced toxicity without compromise on the treatment outcomes [8, 9]. On the other hand, because a relatively longer treatment time is required for STI, accurate patient immobilization becomes imperative for the success of the treatment. Although the initial patient setup error (SE) can be corrected by using a volumetric image and a six degrees of freedom (6-DoF) couch, the patient position during treatment delivery should remain unchanged. In this regard, several studies have reported that the use of a bite block (BB) in conjunction with the normal immobilization devices improves the accuracy of patient setup [10–12]. However, the use of the BB carries the risk of buccal secretion adhering to the immobilization system, which can lead to indirect contact transmission between patients and medical staff. Moreover, droplet transmission may also occur when a patient detaches the medical mask during the setup procedure.

Against this backdrop, in this study, we conducted treatment simulations and procedures with patients wearing medical masks. The intra-fractional patient SE (intra-SE) with and without the BB during STI treatment was compared for patients with brain metastasis.

## MATERIALS AND METHODS

### Patients and simulations

**Fig. 1.**
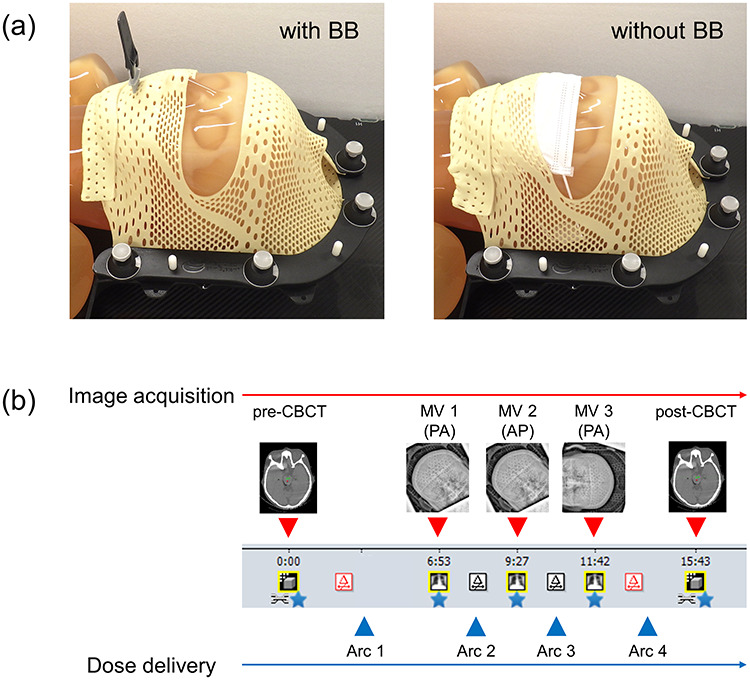
(**a**) Clam-shell-style thermoplastic masks with and without BB, and (**b**) workflow of image acquisition and STI dose delivery. The megavoltage images (MV 1 and MV 3) were acquired at a gantry angle of 179.9° (PA direction), while MV 2 was 0° (AP direction).

This retrospective study, approved by the ethics committee of our institution, included 30 patients with brain metastases who underwent fractionated STI; Table 1 lists the patient details. In the simulations, all patients were immobilized by means of the QFix Encompass SRS immobilization system (Avondale, PA, USA), which consists of a couch insert and a clam-shell-style thermoplastic mask [13]. The Encompass mask consists of anterior and posterior portions, and it can exactly conform to the shape of each patient’s head region to minimize patient motion during the treatment. For a randomly selected 15 patients, who received fractionated STI until the end of 2019, the medical mask was detached during simulation, and a BB was used (Figure 1a, left panel); these patients were designated as belonging to the ‘with-BB’ group. To maintain the patient position during treatment, the thermoplastic mask was extended to shape the nose region as well. For a randomly selected 15 patients, who received fractionated STI from the beginning of 2020, the thermoplastic mask was formed without a BB (‘without-BB’ group), and patients wore medical masks (Figure 1a, right panel) to avoid the risk of interpersonal infection; the nose region was not covered by the thermoplastic mask. In accordance with the interim guidelines recommended by the WHO, the medical mask used was either a surgical or flat/pleated procedure mask [2]. Computed tomography (CT) scanning was performed by means of a dual-energy CT system (Revolution HD; GE Medical Systems, Milwaukee, WI) with the following scanning parameters: tube voltage of 140/80 kVp; tube current of 550–600 mA; helical pitch of 0.531:1. Images were reconstructed with a slice thickness of 1 mm and a field-of-view (FOV) of 320 mm.

**Table 1:** Patient characteristics

	With BB	Without BB
Number of patients (*n*)	15	15
Male/female (*n*)	9/6	8/7
Age (years), median (range)	66 (44–78)	67 (44–82)
Number of metastases, median (range)	3 (1–16)	1 (1–14)
Treatment plan (*n*)		
Prescriotion dose (30/35 Gy)	11/4	6/9
Number of fractions (3/5 fractions)	11/4	4/11
Beam energy (6X/6X-FFF/10X-FFF)	8/7/0	0/14/1

Abbreviation: FFF, flattening filter-free.

### Treatment planning and dose delivery

First, the acquired CT images were transferred to a treatment planning system (Eclipse; Varian Medical Systems, Palo Alto, CA). A planning target volume (PTV) was generated by the addition of an isotropic 1-mm margin to the gross tumor volume. The prescription dose of 30–35 Gy was delivered to the PTV in 3–5 fractions (Table 1). Automated noncoplanar volumetric modulated arc therapy (named HyperArc) plans [5] were generated. One full or half coplanar arc with a couch rotation of 0° and three half non-coplanar arcs with couch rotations of 315, 45 and 90° (or 270°) were used in the treatment. TrueBeam STx and Edge treatment units with a 6-DoF couch were used for dose delivery (Varian Medical Systems).

Figure 1b shows the workflow of the STI dose delivery applied in this study. For treatment, patients were immobilized in the same manner as in the case of the CT simulation, and cone-beam (CB) CT images (pre-CBCT) were acquired before treatment (slice thickness of 1 mm). The patient position was corrected by using the CBCT and the corresponding planning CT images in six directions (three translational directions: anterior–posterior (AP), superior–inferior (SI) and left–right (LR), and three rotational directions: pitch, roll, and yaw), and doses were delivered with the use of a coplanar arc (couch rotation of 0°). Subsequently, the megavoltage (MV) linac graphy (AP or PA direction) was acquired for each couch angle, and bony registration was performed by using the MV images and the corresponding digitally reconstructed radiographs (DRRs) before dose delivery. After the treatments, the acquisition of CBCT images (post-CBCT) were performed to assess the intra-SE.

### Data analysis

The intra-SE was defined as the difference in the patient’s position between the pre- and post-CBCT in the six directions, and thus, the intra-SE included the patient’s position correction done using the MV images during treatment. Subsequently, the intra-SE without MV correction was simulated by adding the amount of shift done using each MV image. Because the MV images were acquired by using the AP/PA beams, the intra-SE without MV correction was simulated along the SI, LR and yaw directions. The 3D translational intra-SE was calculated as the square root of the sum of squares of the three translational intra-SEs, and the 3D rotational intra-SE was the sum of the three rotational angles. The treatment time (TT) was defined as the amount of time taken from the acquisitions of pre-CBCT to post-CBCT.

For the translational intra-SE, the systematic and random error was defined as the mean and standard deviation (SD) of intra-SE, respectively, for each patient. The statistical values of *Σ* and *σ* were calculated as the SD of the systematic error and the root mean square of random error, respectively. The PTV margin (M) to compensate for the patient’s positional uncertainty during STI treatment was calculated by using the following margin recipe [14]:$M=2.5\Sigma +\beta \sqrt{\sigma^2+{\sigma}_p^2}-\beta{\sigma}_p,$where*σ_p_* (3.2 mm) is the width of the penumbra modelled by a cumulative Gaussian, and *β* (1.64) is the value of the inverse cumulative standard normal distribution at the prescribed PTV minimum dose level.

All statistical analyses were performed by using SPSS software (version 24; IBM, Armonk, NY). The Mann–Whitney *U* test was performed for the statistical measurement of the difference in the absolute intra-SE values between the with- and without-BB cases. Subsequently, the paired Wilcoxon signed-rank test was performed to compare the absolute intra-SE corrected using MV image and that without MV correction. To statistically measure the strength of the linear relationship between the 3D translational/rotational intra-SE and TT, a Spearman rank correlation coefficients analysis was performed. The absolute value of Spearman’s *r_s_* was considered very weak for *r_s_* ≤ 0.2, weak for 0.2 < *r_s_* ≤ 0.4, moderate for 0.4 < *r_s_* ≤ 0.7, and strong for 0.7 < *r_s_*. A two-sided *P* value of < 0.05 was considered to indicate statistical significance.

## RESULTS

**Fig. 2.**
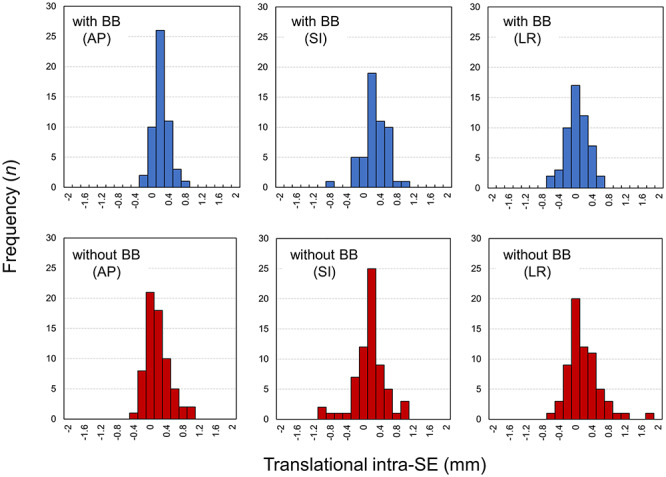
Histograms of translational intra-SE during STI with and without BB.

**Fig. 3.**
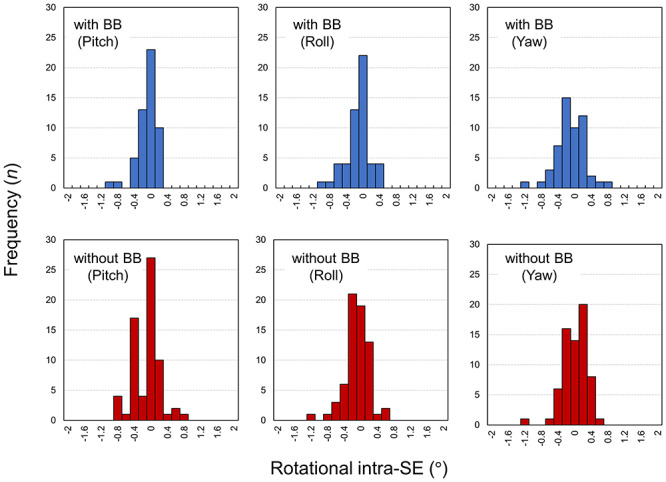
Histograms of rotational intra-SE during STI with and without BB.

A total of 53 and 67 sessions corresponding to the with- and without-BB groups, respectively, were analyzed to assess the intra-SE during STI dose delivery. Figure 2 shows the histograms of the translational intra-SE. A comparable absolute mean (SD) intra-SE is observed in the AP direction (0.19 (0.16) mm vs 0.23 (0.23) mm for the with- and without-BB cases, respectively, *P* = 0.90), in the SI direction (0.30 (0.20) mm vs 0.29 (0.26) mm for the with- and without-BB cases, respectively, *P* = 0.27) and in the LR direction (0.20 (0.16) mm vs 0.29 (0.30) mm for the with- and without-BB cases, respectively, *P* = 0.16). The histograms of the rotational intra-SE are shown in Fig. 3. The respective absolute mean (SD) intra-SEs in the with-BB group are 0.18° (0.20°), 0.23° (0.24°) and 0.27° (0.23°) in the pitch, roll, and yaw directions, respectively. Regarding the without-BB group, the absolute means (SD) are 0.27° (0.25°), 0.23° (0.22°) and 0.22° (0.18°) in the pitch, roll and yaw directions, respectively. There is no statistically significant difference in the rotational intra-SE between the with- and without-BB groups (*P* > 0.10).

**Fig. 4.**
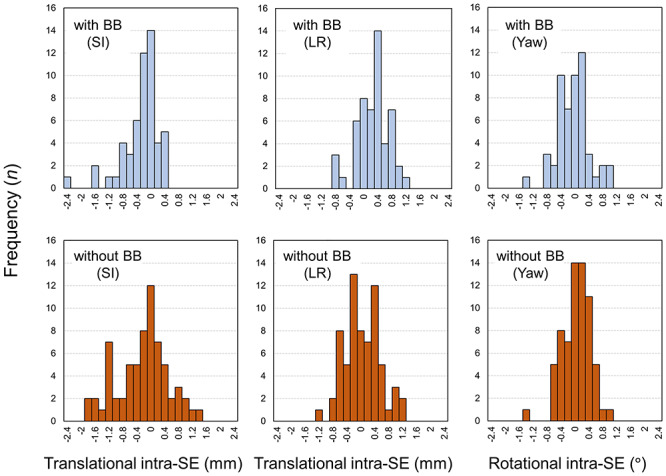
Histograms of translational and rotational intra-SE during STI with and without BB when megavoltage images were not used for patient correction.

Figure 4 illustrates the translational (SI and LR) and rotational (yaw) intra-SEs when the MV images were not used during dose delivery. A relatively wide variation in intra-SE was observed in the SI direction for the without BB group, and the intra-SE was >1 mm for 18 out of 67 sessions, whereas only 5 sessions exceeded an intra-SE of 1 mm in the SI direction for the with BB group. A statistically significant difference in the absolute intra-SE was not observed (*P* > 0.05) between the with and without BB groups (0.43 (0.47) vs 0.6 (0.51) mm, 0.38 (0.28) vs 0.41 (0.29) mm, and 0.34 (0.31) vs 0.31 (0.26) mm in the SI, LR and yaw directions, respectively). In contrast, a significant difference was observed in the SI direction when the intra-SE with MV correction for the with-BB group was compared with that without MV correction (*P* < 0.001). For the without-BB group, the intra-SEs without MV correction were significantly larger in all directions than those with MV correction (*P* < 0.01).

Table 2 lists the systematic and random error for each patient, and the PTV margins calculated to compensate for translational intra-SEs during the STI treatment. For both with and without BB groups, systematic and random errors were <1 mm for all patients. The resultant PTV margins were <1 mm for the with (0.3, 0.4 and 0.5 mm, in the AP, SI and LR directions, respectively) and without (0.5, 0.5 and 0.8 mm, in the AP, SI and LR directions, respectively) BB groups.

**Table 2:** PTV margins compensating for intra-fractional patient setup error

Patient number	With BB						Without BB					
	AP (mm)		SI (mm)		LR (mm)		AP (mm)		SI (mm)		LR (mm)	
	Mean	SD	Mean	SD	Mean	SD	Mean	SD	Mean	SD	Mean	SD
1	0.1	0.1	0.3	0.3	0.0	0.3	0.0	0.1	0.1	0.1	0.3	0.2
2	−0.1	0.1	0.3	0.3	0.2	0.2	0.1	0.0	0.3	0.1	0.0	0.0
3	0.1	0.0	0.3	0.2	0.0	0.1	0.0	0.1	0.3	0.3	−0.2	0.1
4	0.1	0.1	0.3	0.2	0.1	0.1	0.4	0.4	0.1	0.5	0.1	0.1
5	0.3	0.1	0.3	0.4	−0.1	0.0	0.4	0.4	0.0	0.8	0.6	0.2
6	0.2	0.3	−0.1	0.2	0.3	0.1	0.3	0.3	0.0	0.2	−0.2	0.2
7	0.2	0.1	0.0	0.3	−0.2	0.3	−0.1	0.1	0.0	0.1	0.0	0.2
8	0.3	0.1	0.2	0.1	−0.1	0.1	0.0	0.0	0.2	0.1	0.4	0.2
9	0.0	0.1	0.2	0.0	−0.4	0.1	0.1	0.1	0.1	0.1	0.1	0.2
10	0.3	0.1	0.3	0.0	0.1	0.2	0.0	0.2	−0.1	0.1	0.7	0.9
11	0.2	0.1	0.4	0.1	−0.1	0.0	0.4	0.2	−0.2	0.1	0.8	0.2
12	0.1	0.1	0.1	0.1	0.3	0.2	0.3	0.2	0.4	0.2	−0.1	0.1
13	0.1	0.1	0.5	0.3	0.1	0.1	−0.3	0.2	−0.4	0.3	0.0	0.0
14	0.3	0.3	0.2	0.1	0.1	0.1	0.3	0.2	0.4	0.5	−0.1	0.4
15	0.3	0.2	0.5	0.0	−0.1	0.2	0.4	0.2	0.1	0.0	0.3	0.2
Σ	0.1		0.2		0.2		0.2		0.2		0.3	
σ	0.1		0.2		0.2		0.2		0.3		0.3	
M	0.3		0.4		0.5		0.5		0.5		0.8	

**Fig. 5.**
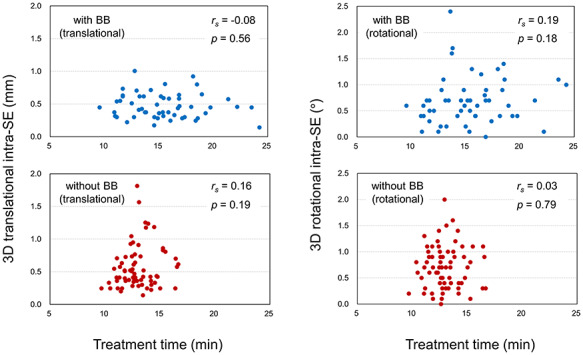
Relationships between 3D intra-SE (translational and rotational) and treatment time.

Figure 5 shows the relationships between the translational and rotational 3D intra-SEs and the treatment time for each group. In the study, because a beam energy of 6 MV with a maximum dose rate of 600 monitor unit/min was used for 8 patients, a longer treatment time (*P* < 0.001) was required for patients of the BB group (mean (SD), 15.4 (3.3) min) than for their without-BB counterparts (mean (SD), 13.0 (1.5) min). There are only ‘very weak’ correlations (|*r_s_*| ≤ 0.2), and no statistically significant correlation is observed between the translational/rotational 3D intra-SE and treatment time (*P* > 0.18).

## DISCUSSION

In this study, we compared the intra-SE for patients immobilized with and without BBs during fractionated STI dose delivery in the context of protecting patients/medical staff from the interpersonal transmission of COVID-19. In this regard, we note that a number of efforts (personal protective equipment, accelerated irradiation schedule, area zoning, terminal disinfection, etc.) aimed at protecting patients and medical staff have been reported specifically for radiation oncology [4, 15–18]. Zhang *et al*. suggested that patients should wear medical masks during treatment to avoid cross infection [16]. They otherwise suggested the use of an additional face shield with the thermoplastic mask. Wei *et al*. attempted a careful balancing of the comfort level of mask wearing against the immobilization accuracy, and they used thermoplastic masks for cranial and head-and-neck patients [4]. They demonstrated that the mean translational and rotational SEs for patients with medical masks were ≤1 mm and ≤ 0.8°, respectively, and no significant difference was observed in relation to patients without medical masks.

Intra-SE during STI may induce non-negligible dosimetric effects, which can induce radiation injury or underdosage for the target volume. In this regard, Roper *et al*. showed that small targets are susceptible to rotational SE during the application of the single-isocentric irradiation technique for multiple targets [19]. Another study by Sagawa *et al*. demonstrated that a 3D rotational SE of ≥2° significantly reduced the target coverage, and this effect was more prominent for tumors located far from the isocenter [20]. Furthermore, for the addition of tight PTV margins (1–2 mm) to the gross tumor volume (GTV) for STI treatment [21–23], the patient SE should be minimized to ensure successful treatment. In this regard, the 6-DoF setup overcomes limitations of the 4-DoF setup in correcting rotational SE, which significantly affects the STI treatment in the case of small targets, small PTV margins, high doses and steep dose gradients [24]. However, maintaining the patient’s position during STI treatment is still challenging in modern radiotherapy.

Previous investigators have reported on the intra-SE during STI dose delivery [12, 25]. In particular, using the thermoplastic mask, Carminucci *et al*. demonstrated that the mean translational intra-SEs during STI were 1.67, 0.73 and 0.75 mm in the AP, SI and LR directions, respectively, whereas the rotational intra-SEs were 0.73, 1.44 and 0.76° in the pitch, roll and yaw directions, respectively [25]. Babic *et al*. compared the intra-SEs among various immobilization systems and found that the thermoplastic mask in conjunction with the BB could stringently limit the intra-SE over the thermoplastic mask alone [12]. In this study, with the aim of protecting patients and medical staff from COVID-19 infections, we firstly compared the intra-SE between the ‘with’ and ‘without-BB’ patients during STI. The mean absolute translational and rotational intra-SE was small (<0.3 mm and <0.3°, respectively) when the BB was not used for patient immobilization, and the intra-SE was comparable with that of patients immobilized using BB (Figs 2 and 3). The resultant PTV margin to compensate for the intra-SE was <1 mm (Table 2). In the STI treatment, tight PTV margins (1–2 mm) are widely adopted to minimize radiation injury to the surrounding normal tissue [26, 27]. Thus, the patient’s position seems to be maintained during the STI treatment, even though the BB was not used for patient immobilization. However, a significantly larger intra-SE was observed when the patient’s position was not corrected using the MV images for both with BB (SI direction) and without BB (SI, LR and yaw directions) groups (Fig. 4). Thus, we need to be aware of the importance of patient correction using MV images especially for the without BB group.

Here, we also mention the limitations of our study. First, the number of patients was limited, and patients were not randomized for either the with- or without-BB categories, which may have resulted in selection bias. In particular, ‘un-flattened’ [flattening filter free (FFF)] beams (6X-FFF and 10X-FFF) can generate a higher dose rate than flattened beams (6X), which can result in a shorter treatment time in the former cases. Second, mechanical uncertainties (such as the kV imager and couch position accuracies) were not considered in the intra-SE obtained using the CBCT images in this study. Third, corrections of the patient’s position during dose delivery (with the use of MV images) were limited to only three directions (SI, LR and yaw); current technologies allow real-time patient motion monitoring in six directions during dose delivery. In this regard, we note that Wiant *et al*. demonstrated that surface imaging offers a non-ionizing, near real-time alternative to radiographic imaging for patient localization during STI [28]. Finally, the PTV margin calculated in the present study could not account for the uncertainties in the mechanical accuracy of the linear accelerator (such as the accuracy of the isocenter, image center and treatment couch movement) [29]. Meanwhile, despite the abovementioned limitations, our quantitative data provide useful information toward protecting against the spread of infections between patients and medical staff during STI treatment.

In conclusion, we found that a PTV margin of <1 mm could be achieved even when patients were immobilized without the BB. That is, the required accuracy for STI can be maintained even without BB when the risk of interpersonal infection is taken into account.

## CONFLICT OF INTEREST

None declared.
